# The Correlation of Serums CCL11, CCL17, CCL26, and CCL27 and Disease Severity in Patients with Urticaria

**DOI:** 10.1155/2016/1381760

**Published:** 2016-01-12

**Authors:** Tao Lu, Xiaoyang Jiao, Mengya Si, Ping He, Jinbo Zou, Shuping Zhang, Kang Zeng

**Affiliations:** ^1^Department of Dermatology, The Affiliated Nanfang Hospital of Southern Medical University, Guangzhou 510515, China; ^2^Department of Dermatology, The First Affiliated Hospital, Shantou University Medical College, Shantou 515041, China; ^3^Cell Biology and Genetics Department, Shantou University Medical College, Shantou 515041, China

## Abstract

*Background.* Chemokines may be involved in the pathogenesis of urticaria, but their correlation with disease severity as well as eruption type is unclear.* Objectives.* The aim of this study was to explore the expression of chemokines in patients with urticaria. The association between disease severity and levels of chemokines was analysed.* Materials and Methods.* Serums CCL11, CCL17, CCL26, and CCL27, D-dimer, C-reactive protein, and total IgE were measured in 51 patients with urticaria and in 25 healthy control subjects.* Results.* Serums CCL11, CCL17, CCL26, and CCL27 were significantly higher in patients with urticaria than in the healthy controls (*P* < 0.05). Serum CCL27 strongly correlated with urticarial disease severity. Serums CCL17, CCL26, and CCL27 significantly correlated with D-dimer, while innercorrelations were noted among the chemokines.* Conclusion.* Our findings reveal that chemokines participate in the pathogenesis of urticaria. Further study in larger cohort is needed to testify whether they could be the biomarkers for predicting the severity of urticaria.

## 1. Introduction

Urticaria is one of the most common inflammatory diseases encountered in routine dermatology practice, characterized by the development of wheals (hives), angioedema, or both [1]. Urticaria consists of acute and chronic subtypes. Acute urticaria (AU) is mostly related to an allergic or pseudoallergic reaction to food, drugs, or infection. AU and angioedema are more frequently associated with identifiable reasons and are often, though not always, related to mast cell and basophil activation caused by several triggers including IgE-mediated and non-IgE-mediated mechanisms. Compared to AU, chronic urticaria is a more complex disease and is less likely to be associated with an identifiable cause, where the trigger is not identifiable in at least 80% to 90% of these patients [2]. Chronic spontaneous urticaria (CSU) occurs as a clinical manifestation of autoimmune causes and patients with CSU show autoantibodies against immunoglobulin E (IgE) itself (anti-IgE) or its high-affinity receptor (anti-Fc*ε*RI). However, autoantibodies are detected in only one-third of the patients, suggesting that other circulating mediators may be involved in the pathophysiology of CSU [3].

Chemokines induce chemotaxis and activation of leukocyte subsets. Chemokines expressed in the skin contribute to the development and maintenance of allergic processes through coordinated recruitment and activation of both leukocytes and residential cells at the site of allergic inflammation [4, 5]. CCL17, expressed by Langerhans cells, endothelial cells, and fibroblasts, is a ligand for CCR4 and CCR8 [6]. The interaction between CCL17 and CCR4 plays a critical role in classical-type activation of macrophages [5]. Serum CCL17 serves as a disease marker for atopic dermatitis [7]. CCL11 and CCL26, expressed by fibroblasts, endothelial cells, and keratinocytes, are a ligand for CCR3. The interaction between CCL26/CCL11 and CCR3 plays an important role in allergic disease in terms of recruiting eosinophils to the site of skin inflammation [8]. CCL27 is expressed only in the skin (esp. in epidermal keratinocytes), which belongs to the CC chemokine family and plays a crucial role in the immune-inflammatory processes. Serum CCL27 concentrations may reflect an overflow of the locally produced chemokine into systemic circulation. Recently, the role of chemokines has been revealed in some skin diseases such as dermatomyositis, vasculitis, and atopic dermatitis [9–12]; however, data regarding the behaviour of chemokines in urticaria are limited [13].

Clinically, the severity of AU and CSU is evaluated by the score scales based on signs and symptoms. However, the evaluation of the intensity of urticaria is difficult as the signs and symptoms can vary significantly over a relatively short period of time. Since the scoring system largely relies on the patient's subjective description of symptoms, it lacks objectivity. Consequently, applying this scoring system to compare the outcomes of clinical trials is a challenge. An additional objective criterion for evaluating the disease severity is necessary [14]. Studies have revealed associations between severity of urticaria and serum biomarkers, including D-dimer [14], C-reactive protein (CRP), total IgE, IL-6, and vascular endothelia growth factor (VEGF) [15] though the validity of these biomarkers remains controversial. Low specificity and sensitivity may be the common drawbacks, leading them inaccurately to reflect the disease severity [16].

Evidence suggests that chemokines may be involved in urticarial pathogenesis, which may influence the severity of urticaria. In this study, we evaluated serum chemokine in patients with AU and CSU and aimed to elucidate whether the expression of chemokines correlated with the eruption type and disease severity of urticaria.

## 2. Methods

The ethics committee of the Shantou University Medical College approved this study and written informed consent was obtained from all the subjects before participation.

### 2.1. Patients

The initial evaluation of the patients was based on history and physical examination. Urticaria activity score (UAS) was calculated in accordance with EAACI/GA2LEN/EDF guidelines [1]. The number of wheals as well as intensity of pruritus was used to estimate UAS on the day of blood sampling. Grading of UAS was made as follows: zero for no wheals, 1 for 1–20 wheals, 2 for 21–50 wheals, and 3 for more than 50 wheals. Pruritus intensity was graded as follows: 0 for no pruritus and 1 for mild, 2 for moderate, and 3 for severe pruritus. UAS (daily total of 0–6) was graded as mild if 0–2, moderate if 3-4, and severe if 5-6. To study the association between expression of chemokines and the disease severity, we regrouped the patients with urticarial severity according to the UAS classification. In order to avoid the influence of small sample size, we combined the AU and CSU groups into one urticarial group.

In combined group, a total of 10 patients were mild, 26 patients were moderate, and 15 patients were severe urticarial group.

CSU is defined as the appearance of wheals and/or angioedema (AE) reoccurring after more than six weeks [1]. Patients were treated with loratadine or cetirizine therapy (10 mg/d, during a period of one week), respectively.

During the follow-up visit, patients were interviewed. Data on age, sex, duration of the disease, distribution of wheal areas on the body, and AE previous or present history of other medical conditions were obtained by direct questioning and physical examination. The patients or control subjects with a history of concurrent autoimmune, inflammatory status (including osteoarthritis and asthma), or infectious disease and patients with physical urticaria or urticarial vasculitis were excluded from the study. Acute urticaria and angioedema are differentiated from CU based on the duration of illness. Urticaria and angioedema with duration of less than 6 weeks is termed acute urticaria. The controls came from healthy individuals with no skin disease and inflammatory diseases, who had not taken any medication for at least 2 weeks prior to the study recruited.

### 2.2. Blood Collection and Measurement

Blood samples were taken from patients before the treatment at their first consultation; however, for CSU patients, blood was taken at three days before blood collection H1-antihistamine drugs were withdrawn. To majority of CSU patients, H1-antihistamine drugs keep efficacy for three days. None of patients took immunosuppressants or any similar drugs during the course of the study. Blood was obtained in the morning during a fasting state, and serum samples were obtained after centrifugation at 1500 ×g at 4°C for 15 min and subsequently stored at −80°C until analysis. Laboratory investigations included CBC and differential count, ESR, as well as serum immunoglobulins (IgG, IgA, and IgM), complement components (C3, C4), CRP, glucose, total IgE, and D-dimer (measured by CA-7000, Sysmex, Japan). The serum total IgE and CRP were measured by ELISA using a commercial kit (Cusabio Biotech Co. Ltd., China). CCL11, CCL17, CCL26, and CCL27 concentrations were measured using Bio-Rad Luminex 200 (Bio-Plex Pro Human Chemokine Assays).

### 2.3. Statistical Analysis

Kruskal-Wallis and Mann-Whitney *U* tests were used for comparisons between groups. Correlations between variables were tested using Spearman's test. Multivariate logistic regression and receiver operating characteristic (ROC) curve analysis were performed to determine the usefulness of the biomarkers for discriminating between urticaria and others. SPSS for Windows version 10.0 was used for statistical analyses (SPSS Incorporated, Chicago, IL, USA). Values of *P* < 0.05 were considered to be statistically significant.

## 3. Results

### 3.1. Demographic and Serum Biomarkers of Patients with AU, CSU, and the Controls (Table 1)

During the study period, a total of 51 patients with urticaria were recruited, of which 27 had AU (6 men, 21 women; median age: 28 years; range: 22.25 to 42 years) and 24 had CSU (5 men, 19 women; median age: 31.5 years; range: 26 to 53.75 years). For comparison, a control group was set, consisting of 25 healthy subjects (12 men, 13 women; median age: 43 years; range: 34.5 to 51.25 years). No significant difference in age was observed among the 3 groups, as well as other parameters such as RBCs, haemoglobin, platelets, and serum glucose. AU and CSU were more frequently seen in middle-aged female patients. WBC quantities in the AU group were significantly higher than in CSU and control (*P* < 0.05), with no difference in WBC found between CSU and control (*P* > 0.05). When we regrouped the patients according to disease severity, WBC quantities of patients with severe urticaria were higher than that in patients with moderate or mild disease, and WBC in moderate urticaria was higher than that in mild disease, though the difference was not significant (*P* > 0.05).

Serum concentrations of immunoglobulins (IgG, IgA, and IgM) and C3/C4 were significantly lower in the AU group than in the CSU group (*P* < 0.05). When we regrouped the urticarial patients according to disease severity, we found that IgG were significantly higher in patients with mild disease than that in patients with moderate-to-severe disease (*P* < 0.05). Similar trend was observed for serum IgM. Patients with mild disease had significantly higher levels of IgM than those with moderate-to-severe disease, and IgM in patients with moderate disease were significantly higher than those in patients with severe disease (*P* < 0.05). On the contrary, IgA levels in patients with mild disease were significantly lower than levels in patients with moderate-to-severe disease (*P* < 0.05). C3 and C4 levels were significantly higher in patients with mild disease than those in patients with moderate-to-severe disease (*P* < 0.05).

### 3.2. Total IgE, CRP, and D-Dimer Concentration and Eosinophil (Eo) Count in Patients with Urticaria and the Control Group (Table 1)

Total IgE concentration was higher in the AU group than that in CSU and control groups, but no significant differences were found (*P* > 0.05). In addition, there was no difference in total IgE levels among patients with various severities (*P* > 0.05).

CRP was significantly higher in the AU group than in the CSU group, and patients with urticaria had significantly higher levels of CRP than those in the healthy controls (*P* < 0.05). CRP levels were higher in patients with moderate-to-severe disease than in patients with mild disease, but there were no significant differences noted (*P* > 0.05). WBC and CRP only indicated the inflammatory response existing in patients with urticaria; they could not distinguish between patients with severe or mild disease.

Patients with AU had significantly higher concentrations of D-dimer than those in CSU and the control groups. D-dimer level was also significantly higher in CSU patients than that in the control group (*P* < 0.05). In patients with various levels of severity, D-dimer was significantly higher in severe patients than in mild-to-moderate patients and was significantly higher in patients with moderate disease than in those with mild disease (*P* < 0.05).

### 3.3. Serum Chemokines in AU, CSU, and Control Groups (Table 1, Figure 1)

Serum concentration of CCL17, CCL26, and CCL27 was significantly higher in the AU group than in the CSU and control groups (*P* < 0.05). CCL17 and CCL26 levels were significantly higher in the CSU group than in the control group (*P* < 0.05). However, there was no difference of serum CCL27 between CSU group and the control group (*P* > 0.05). There was no significant difference in serum concentrations of CCL11 between AU and CSU groups (*P* > 0.05), but CCL11 levels were significantly higher in both AU and CSU groups than that in the control group (*P* < 0.05).

### 3.4. Serum Chemokines in Patients with Different Disease Severity (Table 2, Figure 1)

In order to investigate whether chemokines mirror the disease activity of urticaria, we regrouped 51 patients according to urticarial severity. In order to avoid the influence of small sample size, we combined the AU and CSU groups into one urticarial group. Urticarial patients were divided into 3 groups according to the UAS. CCL27 level was significantly higher in moderate-to-severe patients than that in the mild group (*P* < 0.05). CCL17 and CCL26 levels were significantly higher in patients with moderate-to-severe disease than those in patients with mild disease (*P* < 0.05); however, no difference in CCL17 and CCL26 levels was noted between the moderate and severe groups (*P* > 0.05). In terms of CCL11, there was no difference noted between patients with different levels of severity.

### 3.5. Correlations between Chemokines and Other Biomarkers in AU and CSU Patients (Figure 2)

Spearman rank correlation analysis was performed to evaluate the possible correlations between chemokines and other biomarkers reflecting urticarial severity. Significant correlations were found between the UAS and serum concentration of CCL27 (*r* = 0.354, *P* = 0.01). CCL27 was positively associated with CCL11 (*r* = 0.366, *P* = 0.008), CCL17 (*r* = 0.454, *P* = 0.001), CCL26 (*r* = 0.492, *P* < 0.001), CRP (*r* = 0.602, *P* < 0.001), and D-dimer (*r* = 0.561, *P* < 0.001). CCL17 was positively associated with CCL11 (*r* = 0.402, *P* = 0.005), CCL26 (*r* = 0.540, *P* < 0.001), and D-dimer (*r* = 0.368, *P* = 0.01). CCL11 was positively associated with CCL26 (*r* = 0.332, *P* = 0.019). CCL26 was positively associated with CRP (*r* = 0.490, *P* < 0.001) and D-dimer (*r* = 0.392, *P* = 0.005). Serum chemokines did not significantly correlate with serum total IgE and peripheral Eo (*P* > 0.05).

Weak correlation was found between the UAS and D-dimer level (*r* = 0.292, *P* = 0.04). There was no significant correlation between disease severity and total IgE (*r* = 0.182, *P* = 0.197), CRP (*r* = 0.101, *P* = 0.476), and Eo (*r* = −0.191, *P* = 0.176).

### 3.6. The Diagnostic Significance of Chemokines in the Prediction of Urticaria

To estimate the diagnostic value of chemokines in differentiating between urticarial and nonurticarial patients, multivariate logistic regression and ROC analyses were performed. The ROC established serum chemokine cut-off levels for patients with moderate-to-severe urticaria, as shown in Table 4. The area under the curve (AUC) for D-dimer was 0.922; the sensitivity and specificity of D-dimer were the highest among all parameters studied. The AUC for chemokine was lower than that for D-dimer but higher than that for total IgE and Eo. Among chemokines, CCL26 and CCL27 showed higher AUC (0.907 versus 0.906) values, providing maximum efficiency in prediction of moderate-to-severe urticaria with a sensitivity of 75.0% and 78.8% and specificity of 95% and 90.0%, respectively (Table 3).

## 4. Discussion

Currently, the pathogenesis of urticaria is not well delineated. Treatment is often palliative; therefore, the therapeutic outcome is not optimal. Lack of follow-up and good monitoring biomarkers has led to difficulty in evaluating the efficacy of treatment. Studies have shown that autoimmune mechanisms might play a role in urticaria. Histology of CSU demonstrates a perivascular, nonnecrotizing infiltrate of CD4+ lymphocytes, monocytes, neutrophils, eosinophils, and basophils. Infiltration of inflammatory cells into the skin lesion suggests that histamine is not the sole mediator in the whealing process, supported by the evidence of antihistamine therapy outcomes. Even at high doses, antihistamine is often ineffective, particularly in the severe form of the disease. Recently, it has been proposed that the inflammatory cascade in urticaria may be triggered by an altered chemokine-cytokine network though the mechanism regulating recruitment of inflammatory cells is not completely understood [17]. Chemokines are the main agents inducing chemotaxis and activation of leukocytes, ultimately triggering chemotaxis and transendothelial migration of leukocytes to the site of inflammation [5, 17]. CCL11, CCL17, CCL26, and CCL27 are implicated in the pathogenesis of urticaria [18, 19]. In our study, serum levels of CCL11, CCL17, CCL26, and CCL27 increased significantly in both AU and CSU patients, indicating that elevated CCL11, CCL17, CCL26, and CCL27 are involved in the pathogenesis of urticaria. Mast cell activation leads to the release of chemotactic factors for recruitment of Th2 cells, neutrophils, and eosinophils to the site of inflammation during urticaria, leading to leucocyte (mainly neutrophil and eosinophil) dependent tissue oedema [20]. Both infiltrating inflammatory and endothelial cells have the capacity to deliver chemotactic mediators into the microenvironment. In our study, a close relationship was found between the severity of disease and serum concentration of CCL27, indicating that evaluation of CCL27 is more relevant to the disease severity compared with other chemokines [21]. High concentration of chemokines plays an important role in establishing a microenvironment in which migratory immune cells, together with skin-resident cells, are inducing prolonged inflammation. Both CCL11 and CCL26 play important roles in establishing a Th2-dominant microenvironment in the skin. CCL11 is upregulated in a variety of inflammatory diseases characterized by massive infiltration of eosinophils [5]. In this study, CCL17 is strongly upregulated in moderate-to-severe urticarial patients, especially AU, indicating that elevated CCL17 may be involved in urticaria through playing role in skin homing Th2 cells, classical-type activation of macrophages, and attraction of suppressive T cells to the skin [5, 22, 23]. Quantization of the intensity of urticaria is currently difficult because the signs and symptoms can vary significantly during the disease duration. In this study, we exploited the multivariate logistic regression and ROC analyses to estimate the diagnostic value of chemokines in differentiating the intensity of urticaria. Our data showed that AUC for the chemokines was higher than that of conventional biomarkers (total IgE, CRP, and Eo). Moreover, CCL26 and CCL27 showed a high AUC, providing maximum efficiency in prediction of moderate-to-severe urticaria with high sensitivity and specificity. Therefore, monitoring serum chemokines are valuable in the evaluation of urticarial severity [24–26].

Coagulation factors may enhance vascular permeability or induce mast cell degranulation. Increased D-dimer has been described in patients with CSU and might serve as a marker for CSU with angioedema severity [14, 27]. Recent reports have revealed that D-dimer levels are elevated in patients with active CSU or during urticaria exacerbation and return to normal during remission [28, 29]. Significantly higher levels of D-dimer were found in our study as well, either in AU or in CSU, when compared to the control group. A positive association was observed between the urticarial score and D-dimer concentration, indicating that an elevation of D-dimer contributes to the pathogenesis of urticaria and demarcates the potential role of D-dimer as a biomarker for disease severity [30]. This is further confirmed by the higher sensitivity and specificity for D-dimer compared to other parameters assessed in this study. Our data revealed that measurement of D-dimer can be useful to assess the severity of disease in patients with urticaria. The role of D-dimer may connect IgE with the extrinsic pathway of coagulation [31]. Positive associations were found between D-dimer and CCL27, CCL17, and CCL26 in our study, indicating that D-dimer and chemokine might play a concomitant role in the pathogenesis of urticaria. However, D-dimer was also elevated in some coagulating disease or anticoagulant therapy; the role of D-dimer in urticaria needs to be carefully evaluated.

Until recently, limited laboratory tests were available to exclude underlying causes of urticaria. Routine laboratory testing in patients with AU and CSU, whose history and physical examination reveal a lack of atypical features, rarely yields clinically significant findings [15]. Elevated CRP in the sera of CSU patients reflects the systemic effects of inflammatory mediators associated with the disease, suggesting that CRP is a marker for systemic CSU [32]. However, its relative nonspecificity does not qualify CRP as a specific biomarker (as it may be elevated in a number of inflammatory processes). With regard to urticarial patients, targeted laboratory testing based on clinical suspicion is appropriate, but skin or* in vitro* testing for IgE in response to inhalants or foods and/or extensive laboratory testing are not recommended as these are not cost-effective and do not result in improved patient care outcomes [14].

Biologics targeting chemokines and their receptors are promising strategies for treatment of various skin diseases that are resistant to the currently available therapeutic options [5]. However, there is still a need to define the diagnostic criteria and develop reliable diagnostic tests to identify these patients [33]. So far, data about chemokines and severity of urticaria is scarce. Our study indicates a close relationship between serums CCL11, CCL17, CCL26, and CCL27 and the severity of disease of patients with AU and CSU. To testify whether CCL11, CCL17, CCL26, and CCL27 are to be a useful indicator of urticaria disease severity, further study on larger cohort is needed.

There are two major limitations of the study. The first is that the study did not compare patients during active disease and during remission. The second is low samples size; more reliable data on chemokines may be got from larger cohort.

## Figures and Tables

**Figure 1:**
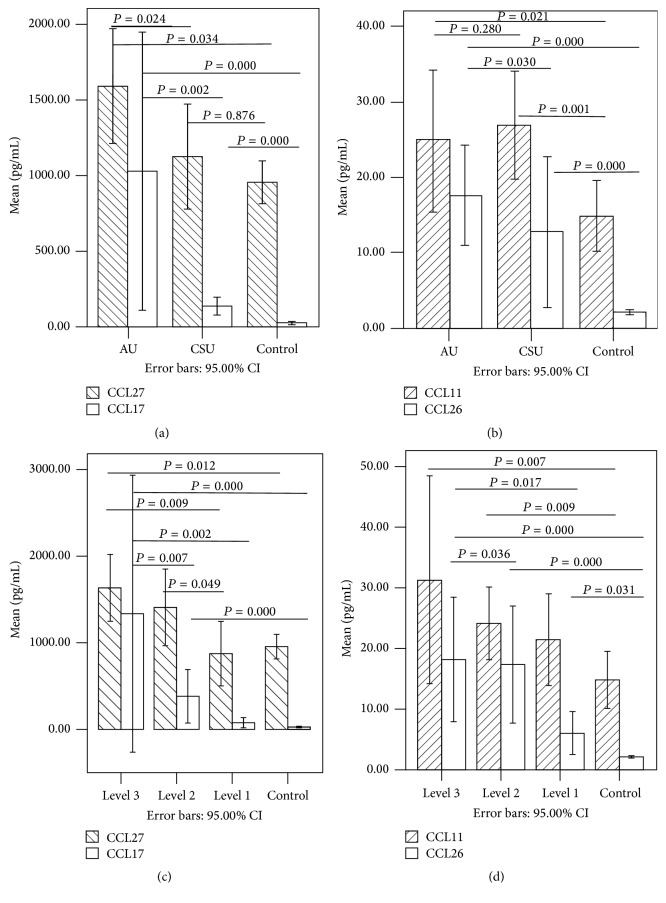
Levels of CCL27 and CCL17 in AU, CSU, and control group (a). Levels of CCL11 and CCL26 in AU, CSU, and control group (b). Levels of CCL27 and CCL17 in different severity and control group (c). Levels of CCL11 and CCL26 in different severity and control group (d).

**Figure 2:**
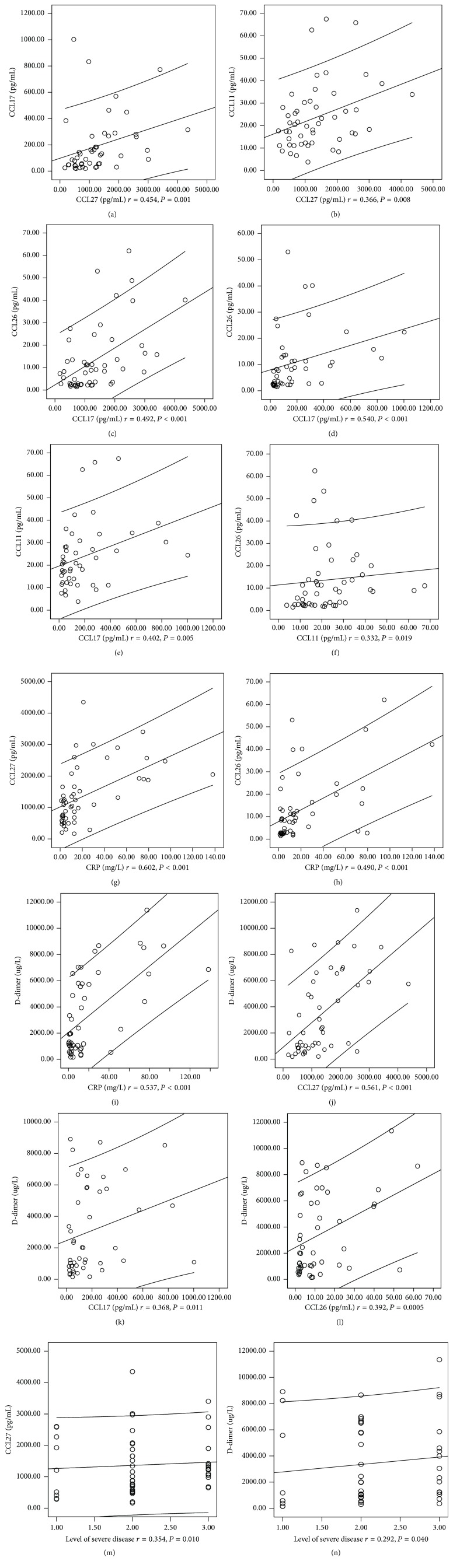
Correlations existed among chemokine biomarker and conventional parameters. Positive correlations were observed between CCL27 and CCL11 (a), between CCL27 and CCL11 (b), between CCL27 and CCL26 (c), between CCL17 and CCL26 (d), between CCL17 and CCL11 (e), between CCL11 and CCL26 (f), between CCL27 and CRP (g), between CCL26 and CRP (h), between CRP and D-dimer (i), between CCL27 and D-dimer (j), between CCL17 and D-dimer (k), between CCL26 and D-dimer (l), between CCL27 and levels of severe disease (m), and between D-dimer and levels of severe disease (n).

**Table 1:** General characteristic of AU, CSU, and control groups.

Variables	AU (*n* = 28)	CSU (*n* = 24)	Control (*n* = 25)
Age (year)	28.00 (22.25–42.00)	31.50 (26.00–53.75)	43.00 (34.50–51.25)
Gender (male/female)	6/22	5/19	12/13
CCL27 (pg/mL)	1281.23 (987.27–2069.86)	754.94 (516.88–1659.07)^*∗*^	976.54 (724.25–1142.84)^♦^
CCL11 (pg/mL)	17.56 (11.85–33.11)	22.70 (15.86–28.21)	12.84 (9.00–17.78)^♦ΔΔ^
CCL26 (pg/mL)	11.91 (3.72–24.18)	4.57 (2.45–11.16)^*∗*^	2.00 (1.74–2.34)^♦♦ΔΔ^
CCL17 (pg/mL)	159.92 (86.84–722.18)	67.55 (33.19–241.44)^*∗∗*^	21.29 (16.37–34.62)^♦♦ΔΔ^
D-dimer (ug/L)	4550.00 (1122.50–6955.00)	1105.00 (642.50–5017.50)^*∗∗*^	245.00 (160.00–487.50)^♦♦ΔΔ^
IgE (ng/mL)	530.88 (309.91–746.35)	442.22 (372.18–548.56)	440.70 (379.15–605.16)
IgG (g/L)	10.47 (9.89–11.05)	11.80 (11.10–12.50)^*∗∗*^	11.56 (7.51–15.60)
IgA (g/L)	1.97 (1.79–2.15)	2.14 (1.94–2.34)^*∗∗*^	2.68 (0.82–4.53)
IgM (g/L)	1.12 (1.02–1.22)	1.51 (1.31–1.71)^*∗∗*^	1.75 (0.46–3.04)
C3 (g/L)	0.89 (0.84–0.94)	0.99 (0.92–1.06)^*∗∗*^	1.16 (0.79–1.52)
C4 (g/L)	0.19 (0.17–0.21)	0.21 (0.19–0.23)^*∗∗*^	0.27 (0.16–0.38)
ESR (mm/h)	16.00 (13.30–18.70)	12.46 (9.83–15.10)^*∗∗*^	7.50 (0.50–15.00)
WBC (10*E* + 9/L)	11.05 (8.52–13.31)	7.53 (6.15–9.06)^*∗∗*^	6.90 (6.15–7.63)^♦♦^
Eo (10*E* + 9/L)	0.06 (0.02–0.16)	0.09 (0.04–0.15)	0.16 (0.09–0.28)^♦♦Δ^
RBC (10*E* + 12/L)	4.40 (4.04–4.62)	4.44 (4.16–4.93)	4.74 (4.52–5.26)^♦♦^
Hb (g/L)	126.00 (116.25–135.50)	131.00 (125.50–140.75)	142.50 (133.00–152.50)^♦♦Δ^
PLT (10*E* + 9/L)	250.50 (193.25–305.25)	245.50 (226.50–319.75)	239.50 (217.75–261.50)^Δ^
Glu (mmol/L)	5.41 (4.74–5.64)	5.27 (4.89–5.64)	5.15 (5.03–5.36)
CRP (mg/L)	29.86 (15.85–43.87)	12.83 (6.01–19.65)	4.00 (0.01–8.00)^♦Δ^

CCL, chemokine ligand; Ig, immunoglobulin; C3, complement 3; C4, complement 4; ESR, erythrocyte sedimentation rate; WBC, white blood cell; Eo, eosinophil; RBC, red blood cell; PLT, platelet; Glu, glucose; CRP, C-reactive protein; AU, acute urticaria; CSU, chronic spontaneous urticaria.

^*∗*^ *P* < 0.05 and ^*∗∗*^ *P* < 0.01 when AU group was compared with CSU group.

^*♦*^ *P* < 0.05 and ^*♦♦*^ *P* < 0.01 when AU group was compared with the control.

^Δ^ *P* < 0.05 and ^ΔΔ^ *P* < 0.01 when CSU group was compared with the control.

**Table 2:** General characteristic and biomarkers in patients with various severities.

Variables	Severe patients (*n* = 15)	Moderate patients (*n* = 26)	Mild patients (*n* = 10)
Age (year)	34.50 (22.00–46.50)	28.00 (24.00–42.00)	31.50 (23.00–45.00)
Gender (male/female)	4/11	7/19	0/10
CCL27_Serum_ (pg/mL)	1134.80 (1088.99–2273.87)	1183.00 (510.08–2048.48)^Δ^	631.4 (521.35–1049.26)^♦♦^
CCL11_Serum_ (pg/mL)	18.84 (13.10–39.31)	23.85 (11.70–28.25)	20.12 (15.40–23.76)
CCL26_Serum_ (pg/mL)	11.08 (5.24–22.28)	8.55 (2.96–22.51)	3.03 (2.27–11.23)^*♦∗*^
CCL17_Serum_ (pg/mL)	140.08 (57.29–648.21)	171.79 (57.00–314.95)	35.02 (22.28–100.79)^*♦♦∗∗*^
D-dimer_Plasma_ (ug/L)	3865.00 (1235.00–7820.00)	2225.00 (850.00–65510.00)	935.00 (580.00–3360.00)^♦^
IgE_Serum_ (ng/mL)	530.88 (429.76–743.62)	457.08 (326.90–602.43)	442.92 (289.52–705.80)
IgG_Serum_ (g/L)	11.06 (10.94–11.06)	10.86 (10.58–10.86)^Δ^	12.63 (12.20–12.63)^*♦∗∗*^
IgA_Serum_ (g/L)	2.15 (2.15–2.15)	2.05 (1.88–2.05)^Δ^	1.93 (1.93–2.04)^*♦♦∗*^
IgM_Serum_ (g/L)	1.13 (1.13–1.13)	1.40 (1.21–1.40)^ΔΔ^	1.43 (1.43–1.51)^*♦♦∗*^
C3_Serum_ (g/L)	0.84 (0.83–0.84)	0.97 (0.90–0.97)^ΔΔ^	1.01 (0.95–1.11)^*♦♦∗*^
C4_Serum_ (g/L)	0.20 (0.20–0.21)	0.20 (0.18–0.20)	0.21 (0.20–0.22)^*∗*^
ESC (mm/h)	13.25 (3.37–46.55)	11.55 (3.25–27.60)^ΔΔ^	7.10 (2.07–13.50)^♦♦^
CRP (mg/L)	12.00 (8.50–12.75)	17.67 (12.75–17.75)	7.25 (7.19–7.44)
WBC_Blood_ (10*E* + 9/L)	11.49 (8.02–13.15)	8.99 (6.37–10.62)	7.55 (6.24–10.12)^♦^
Eo_Blood_ (10*E* + 9/L)	0.06 (0.01–0.10)	0.07 (0.03–0.15)	0.10 (0.04–0.14)
RBC_Blood_ (10*E* + 12/L)	4.47 (4.05–4.73)	4.40 (4.14–4.54)	4.32 (4.05–4.94)
Hb_Blood_ (g/L)	126.00 (113.50–136.50)	130.00 (125.00–135.00)	126.50 (115.00–141.00)
PLT_Blood_ (10*E* + 9/L)	235.00 (193.50–303.50)	244.00 (209.00–308.00)	269.50 (243.00–320.00)
Glu_Serum_ (mmol/L)	5.39 (4.84–5.67)	5.28 (4.84–5.57)	5.13 (4.90–5.65)

CCL, chemokine ligand; Ig, immunoglobulin; C3, complement 3; C4, complement 4; ESR, erythrocyte sedimentation rate; WBC, white blood cell; Eo, eosinophil; RBC, red blood cell; PLT, platelet; Glu, glucose; CRP, C-reactive protein.

^Δ^ *P* < 0.05 and ^ΔΔ^ *P* < 0.01 when severe group compared with moderate group.

^*♦*^ *P* < 0.05 and ^*♦♦*^ *P* < 0.01 when severe group compared with mild group.

^*∗*^ *P* < 0.05 and ^*∗∗*^ *P* < 0.01 when moderate group compared with mild group.

**Table 3:** Correlations among the chemokines, D-dimer, IgE, and CRP.

Variables	CCL27	CCL17	CCL11	CCL26	D-dimer	IgE	Eo	CRP
CCL17	*r* = 0.454							
	*P* = 0.001^*∗∗*^							

CCL11	*r* = 0.366	*r* = 0.402						
	*P* = 0.008^*∗∗*^	*P* = 0.005^*∗∗*^						

CCL26	*r* = 0.492	*r* = 0.540	*r* = 0.332					
	*P* < 0.001^*∗∗*^	*P* < 0.001^*∗∗*^	*P* = 0.019^*∗*^					

D-dimer	*r* = 0.561	*r* = 0.368	*r* = −0.078	*r* = 0.392				
	*P* < 0.001^*∗∗*^	*P* = 0.011^*∗*^	*P* = 0.594	*P* = 0.005^*∗∗*^				

IgE	*r* = −0.081	*r* = −0.009	*r* = −0.228	*r* = −0.137	*r* = 0.117			
	*P* = 0.568	*P* = 0.950	*P* = 0.108	*P* = 0.338	*P* = 0.418			

Eo	*r* = 0.185	*r* = −0.109	*r* = 0.226	*r* = 0.104	*r* = −0.011	*r* = −0.238		
	*P* = 0.190	*P* = 0.460	*P* = 0.107	*P* = 0.468	*P* = 0.938	*P* = 0.089		

CRP	*r* = 0.602	*r* = 0.272	*r* = 0.105	*r* = 0.490	*r* = 0.537	*r* = 0.064	*r* = 0.013	
	*P* < 0.001^*∗∗*^	*P* = 0.062	*P* = 0.464	*P* < 0.001^*∗∗*^	*P* < 0.001^*∗∗*^	*P* = 0.650	*P* = 0.926	

Level of severe disease	*r* = 0.354	*r* = 0.218	*r* = 0.008	*r* = 0.255	*r* = 0.292	*r* = 0.182	*r* = −0.191	*r* = 0.101
	*P* = 0.010^*∗*^	*P* = 0.137	*P* = 0.957	*P* = 0.071	*P* = 0.040^*∗*^	*P* = 0.197	*P* = 0.176	*P* = 0.476

^*∗*^ *P* < 0.05 and ^*∗∗*^ *P* < 0.01.

**Table 4:** Receiver operating characteristic curve analysis of chemokines in urticaria group.

Variables	AUC	*P*	Cut-off value	Sensitivity	Specificity	PPV	NPV
D-dimer	0.922	<0.001	775.00	80.8	95.0	97.7	70.6
CCL26	0.907	<0.001	2.75	75.0	95.0	95.1	63.9
CCL17	0.906	<0.001	47.41	78.8	90.0	93.2	66.7
CCL11	0.725	0.003	19.33	53.8	90.0	93.3	48.9
CCL27	0.589	0.242	1123.89	51.9	80.0	84.4	44.4
IgE	0.542	0.580	430.51	61.5	55.0	74.1	41.2
Eo	0.225	0.000	0.57	1.9	95.0	50.0	32.0

AUC: areas under curve. PPV: positive predictive value. NPV: negative predictive value.
